# ABNet: AI-Empowered Abnormal Action Recognition Method for Laboratory Mouse Behavior

**DOI:** 10.3390/bioengineering11090930

**Published:** 2024-09-17

**Authors:** Yuming Chen, Chaopeng Guo, Yue Han, Shuang Hao, Jie Song

**Affiliations:** 1 Software College, Northeastern University, Shenyang 110169, China; 2290126@stu.neu.edu.cn (Y.C.); guochaopeng@swc.neu.edu.cn (C.G.); 2 College of Life and Health Sciences, Northeastern University, Shenyang 110169, China; 2101350@stu.neu.edu.cn

**Keywords:** mice, mouse behavior, action recognition, semi-supervised learning, computer vision

## Abstract

The automatic recognition and quantitative analysis of abnormal behavior in mice play a crucial role in behavioral observation experiments in neuroscience, pharmacology, and toxicology. Due to the challenging definition of abnormal behavior and difficulty in collecting training samples, directly applying behavior recognition methods to identify abnormal behavior is often infeasible. This paper proposes ABNet, an AI-empowered abnormal action recognition approach for mice. ABNet utilizes an enhanced Spatio-Temporal Graph Convolutional Network (ST-GCN) as an encoder; ST-GCN combines graph convolution and temporal convolution to efficiently capture and analyze spatio-temporal dynamic features in graph-structured data, making it suitable for complex tasks such as action recognition and traffic prediction. ABNet trains the encoding network with normal behavior samples, then employs unsupervised clustering to identify abnormal behavior in mice. Compared to the original ST-GCN network, the method significantly enhances the capabilities of feature extraction and encoding. We conduct comprehensive experiments on the Kinetics-Skeleton dataset and the mouse behavior dataset to evaluate and validate the performance of ABNet in behavior recognition and abnormal motion detection. In the behavior recognition experiments conducted on the Kinetics-Skeleton dataset, ABNet achieves an accuracy of 32.7% for the top one and 55.2% for the top five. Moreover, in the abnormal behavior analysis experiments conducted on the mouse behavior dataset, ABNet achieves an average accuracy of 83.1%.

## 1. Introduction

With the rapid development of neurosciences, pharmacology, toxicology, pathology, and psychology, the demand for quantitative analysis of abnormal behavior in experimental animals is steadily increasing [1,2,3,4,5]. Laboratory mice, as representatives of rodent species, share similar genomic sequences with humans [6]. Their small size, low cost, and ease of reproduction make them increasingly crucial in behavioral research [7].

Traditional approaches to abnormal mouse behavior recognition primarily utilize sensor technology and biomarker analysis. Sensor technology applies various sensors to monitor physiological indices or motion parameters of mice to capture abnormal changes in behavior. However, this method heavily depends on the accuracy of sensors, which may be influenced by noise, drift, and interference, leading to data errors and instability [8]. In some cases, sensors must be implanted or attached to mice, potentially interfering with their behavior and physiological state, thereby affecting the natural manifestation of behaviors [9]. Biomarker analysis methods infer behavioral states and abnormalities in mice by analyzing biomarkers in mouse body fluids, tissues, or blood [10]. Nevertheless, collecting these samples requires specific operation techniques and complex experimental equipment. The use of such techniques and equipment may be restricted by factors such as technical proficiency, resource limitations, and time costs.

In recent years, the rapid development of artificial intelligence (AI) has significantly improved research efficiency and reduced labor and material costs when combined with biological research. Therefore, the automated recognition and analysis of abnormal mouse behavior is paramount. This not only accelerates research progress but also helps to understand the genetic and environmental factors influencing mouse behavior more profoundly, serving as a valuable tool in drug development and disease modeling [11,12].

Currently, the main directions for AI-based abnormal mouse behavior recognition are video-based methods and skeleton-based methods. Video-based recognition typically considers mice as single tracking points or uses image processing techniques to extract mouse contours as body posture features, facilitating the detection and classification of abnormal behaviors [13]. For instance, in LabGym, accurate behavior recognition of animal movement patterns is achieved by continuously extracting peripheral hubs to form “pattern images” [14]. AlphaTracker primarily obtains movement information by tracking multiple markers on mice and performs behavior recognition based on this information [15]. However, these methods often struggle to accurately depict the fine-grained motion characteristics of mice [16]. Although skeleton-based methods can provide more detailed motion information [17,18], over-reliance on prior skeletal knowledge may limit detection performance. Additionally, pose estimation methods in mouse skeletal recognition are primarily transferred from human pose recognition, yet there are differences between human and mouse skeletons [19]. As a result, the adopted pose estimation algorithms may achieve a different performance level than in human skeleton detection, and inaccurate pose estimations can affect the validity of abnormal behavior recognition.

Moreover, there are additional challenges in the automated recognition of abnormal mouse behavior. Abnormal mouse behaviors are difficult to define precisely, and data samples are hard to collect. Therefore, when recognizing abnormal behaviors through manually defined behavior quantification rules or training behavior classifiers, models may need help to learn sufficient features of abnormal behavior [20]. Consequently, the detection results may need improvement.

**Figure 1:**
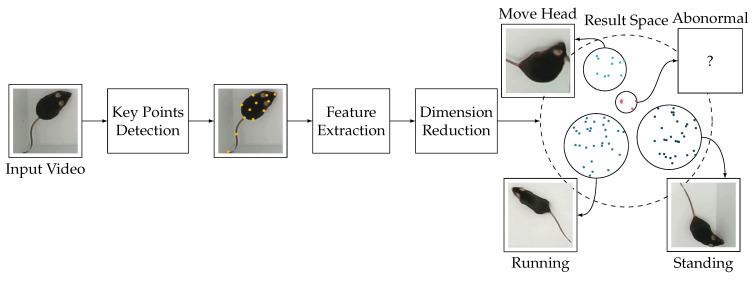
ABNet overall process.

To address these challenges, we propose a novel approach named ABNet for recognizing abnormal mouse behavior. The overall process of ABNet is shown in Figure 1. First, we extract key points of the mouse skeleton using a pose recognition algorithm to form a skeletal sequence. Next, we use a motion feature extraction and encoding network to extract and encode motion features from the skeletal sequence, forming high-dimensional feature information. Finally, we perform dimensionality reduction on these features and conduct clustering, identifying abnormal behavior based on the characteristic that abnormal samples can be isolated with fewer random feature divisions than normal samples.

ABNet does not rely on abnormal behavior samples to train the feature extraction network and behavior classifier; instead, it uses only normal behavior samples to train the motion information extraction and encoding network, thereby avoiding poor detection results due to the limited availability of abnormal samples. ABNet employs an enhanced ST-GCN [21] as the motion information extraction and encoding network. In this network, Gaussian noise perturbation is applied to the prior matrix of key points, creating links between adjacent key points to achieve a better spatial structure for describing motion features. By introducing a channel attention mechanism in the ST-GCN, the model is better able to focus on feature channels with high-quality information and suppress the impact of low-quality information channels on model performance. The original ST-GCN used 1 × 1 convolutions with a kernel size of 9 for modeling in the temporal dimension, which led to computational and parameter redundancy. By replacing the large kernel convolutions with a multi-branch temporal convolutional network comprising convolution, pooling layers, and 1D convolutions, we effectively improve the temporal modeling capability of the ST-GCN.

The main contributions and innovations of this study are outlined as follows:

- We deploy a fine-tuned DeepLabCut model to detect key points in mouse skeletons. Visual comparison shows that this model significantly outperforms object detection methods in capturing detailed keypoint information. This superior performance establishes a robust foundation for the accurate recognition of abnormal behaviors.
- We propose and utilize an improved ST-GCN model to extract and encode motion feature information in mouse skeletal sequences. Ablation experiments demonstrate that, compared to the original ST-GCN, the addition of Gaussian noise, SENet channel attention mechanisms, and a multi-branch TCN structure effectively enhances the model’s feature extraction and encoding capabilities.
- We propose a semi-supervised learning method for abnormal mouse behavior recognition, denoted as ABNet. This method requires only accurately defined normal behavior samples to train the enhanced ST-GCN, thereby establishing a normal behavior encoding model. By subsequently applying clustering techniques, it can effectively detect abnormal behavior. Comparative experiments with other methods demonstrate the effectiveness of this approach.

The remainder of this paper is organized as follows. Section 2 offers an overview of relevant work in multi-object pose estimation, action recognition, and abnormal behavior recognition. In Section 3, we provide a detailed account of our keypoint detection methodology. Section 4 elucidates the feature extraction method based on an improved ST-GCN, while Section 5 proposes our behavior analysis method, encompassing both behavior classification and abnormal behavior detection. A series of experimental designs, corresponding results, and analyses are provided in Section 6 to demonstrate the effectiveness of our methodology. Section 7 summarizes our proposed method and the resulting experimental outcomes. Finally, Section 8 discusses our future work.

## 2. Related Works

This section provides an overview of the related work in the field of abnormal action recognition, focusing on multi-object pose estimation, action recognition, and anomaly behavior recognition.

### 2.1. Multi-Objective Pose Estimation

Multi-object pose estimation refers to the task of simultaneously estimating the poses of multiple objects in an image or a sequence of images. It involves detecting and localizing multiple objects in the image and accurately estimating their respective poses, including their positions, orientations, and shapes. Multi-object pose estimation can be categorized into the following two approaches based on the detection process: top-down and bottom-up approaches. In the top-down approach, the idea is, first, to detect all the objects in the input image, then estimate the pose information for each individual object [22]. In contrast, the bottom-up approach detects all the pose information in the input image, then assigns the pose information to different detected objects using predefined association rules [23]. Both approaches inevitably need to consider how to address issues such as occlusion and overlap between key points [24].

DeepCut is an earlier bottom-up approach for multi-object pose estimation. The algorithm introduces a method that jointly solves object detection and pose estimation [23]. It uses convolutional neural networks to detect skeletal key points in the input image and combines them into density maps. Then, it utilizes object detection algorithms to locate the positions of the objects and associates the key points with different objects by linking the position information and the density maps.

Building upon the principles of DeepCut, Mathis et al. proposed DeepLabCut [25]. DeepLabCut leverages deep neural networks and transfer learning techniques to train models for pose estimation without markers. It offers a user-friendly interface and extensive pre-training on large-scale datasets such as ImageNet [26]. Additionally, DeepLabCut has been tested and calibrated across different species, including mice and fruit flies, allowing the algorithm to achieve performance similar to human behavior detection with minimal data samples. Therefore, we chose DeepLabCut as the foundational model for pose estimation and behavioral analysis.

### 2.2. Action Recognition

Action recognition aims to identify different actions performed by targets from a sequence of video frames, such as walking, running, and standing. Deep learning-based methods for action recognition mainly consist of the following three approaches: single-stream, two-stream, and skeleton-based approaches. The single-stream approach employs a single network, while the two-stream approach utilizes two networks to extract spatial and temporal information separately. The skeleton-based method utilizes skeleton structure data for recognition. Skeleton-based methods differ based on the data, comprising (1) those treating skeleton structure data as a time series, mapping the data into coordinate vector sequences based on the sequential nature of skeleton key points and using recurrent neural networks (RNNs) for behavior recognition [27,28]; (2) those mapping the skeleton structure data into pseudo-images and using convolutional neural networks for behavior recognition [29,30,31]; and (3) those leveraging the natural topological structure of the skeleton structure and combining spatial and temporal data for behavior recognition [21,31,32,33,34].

However, skeleton structure data differ from video or text data, and directly mapping them into coordinate vector sequences or pseudo-images inevitably results in the loss of their inherent information. The skeleton structure possesses a natural topological structure that aligns well with graph data representation. Mapping the key points in the skeleton structure to vertices in a graph structure and connecting the key points with edges in the graph better express the information contained in the skeleton structure data. Based on this concept, Yan et al. proposed a spatio-temporal graph convolutional network (ST-GCN) that directly models skeleton data as a graph structure [21,32,35,36,37,38].

Although the ST-GCN network can accurately encode motion trajectories, the original ST-GCN also has certain limitations. First, the adjacency matrix of ST-GCN is manually predefined, leading to a relatively rigid structure that hampers the capture of correlations between non-adjacent key points. Secondly, ST-GCN neglects the importance of channels and the relationships among them, which can affect the network’s performance. We improve ST-GCN by incorporating a Gaussian noise matrix and attention mechanism to address these issues. This enables the network to focus more on key channel features, significantly enhancing its feature extraction and encoding capabilities.

### 2.3. Anomaly Behavior Recognition

Anomaly behavior recognition is a task within action recognition that can be categorized into probability models, reconstruction models, and distance models based on different data processing methods.

Probability models evaluate the normal probability of behavior data to determine anomalies, utilizing techniques such as Gaussian mixture models, multivariate Gaussian models, and autoregressive equations to learn probability distributions [39]. While probability models offer a comprehensive theoretical framework, accurately learning probability distributions is challenging, especially with high-dimensional data, leading to limited effectiveness.

Reconstruction models determine anomalies by measuring the error between reconstructed behavior and normal behavior, employing generative adversarial networks (GANs) and autoencoders. For instance, one approach involves training distinct GANs for different features and combining the generated errors to identify anomalies [40]. Another approach combines sparse coding with stacked recurrent neural networks, encoding adjacent video frames and utilizing reconstruction errors for anomaly detection [41]. However, reconstruction models face difficulty distinguishing normal and anomalous behavior, as similar anomalies can be reconstructed.

Distance models determine anomalies by measuring the distance between behavior data and normal behavior using support vector machines (SVMs) and clustering algorithms. For example, the GEPC algorithm maps skeleton key points to other feature spaces for clustering-based anomaly detection [42]. Another approach involves utilizing a convolutional autoencoder to encode appearance and motion features into distances, enabling unsupervised anomaly detection [43].

We propose ABNet based on a distance model, which focuses on improving the quality of feature information when computing distances. Obtaining high-quality feature information by directly mapping skeletal key points is challenging. Our approach involves training an enhanced ST-GCN network using normal behavior data, enabling the extraction and encoding of motion information contained in skeletal keypoint data. The computed distances are then based on the feature information output by this network. Compared to direct mapping, this method effectively extracts temporal and spatial features from skeletal key points, enhancing the quality of information.

## 3. Keypoint Detection

**Figure 2:**
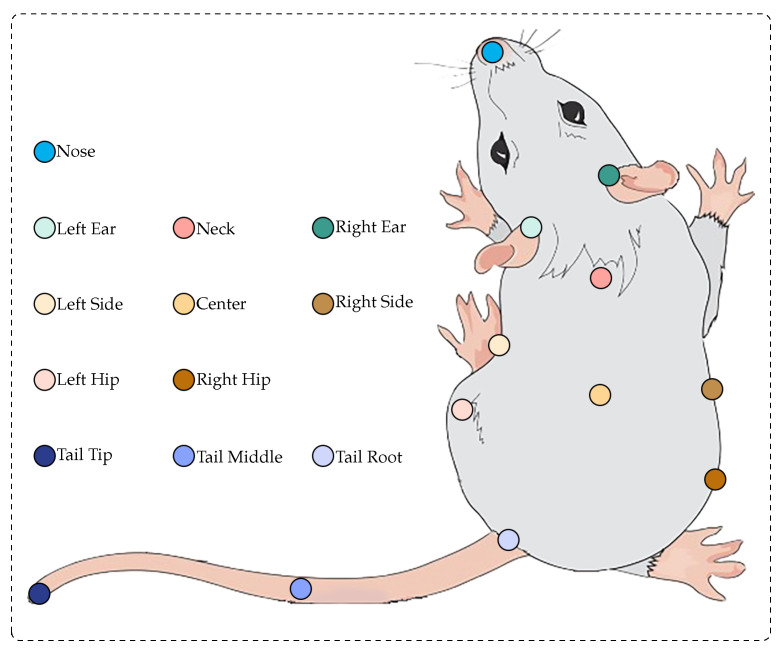
Skeleton points of a mouse.

ABNet utilizes 12 key points representing the skeletal structures of mice as inputs for action recognition, shown in Figure 2. To extract these key points, we apply DeepLabCut [25], an open-source software toolbox designed for 2D and 3D pose estimation in behavioral experiments. Processing and training DeepLabCut with our data enables fast and accurate keypoint detection.

This keypoint mapping is crucial for recognizing mouse posture and behavior. Due to its superior performance, DeepLabCut was chosen as ABNet’s keypoint detector. Unlike traditional object detection frameworks such as YOLOv9, which are optimized for bounding-box predictions, DeepLabCut focuses specifically on the localization of individual body landmarks. This specialization allows for higher accuracy in keypoint detection.

**Figure 3:**
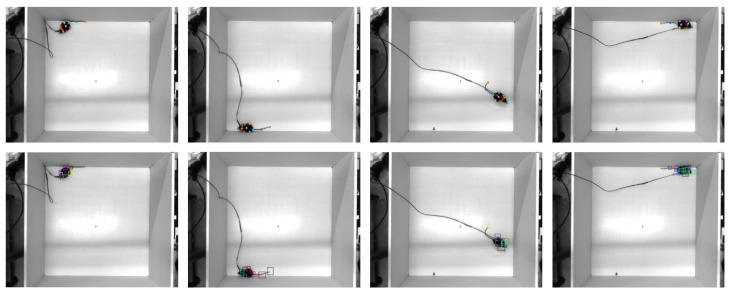
Comparison of keypoint detection between DeepLabCut and YOLOv9. The first line is the detection results of the DeepLabCut pose estimation algorithm, and the second line is the detection results of the YOLOv9 object detection algorithm.

In our experiments, we conducted visual comparisons between DeepLabCut and YOLOv9. The results showed that YOLOv9 tends to miss certain key points, leading to incomplete detections. This problem of missing detections is more pronounced when handling finer, more dynamic key points, such as those in the mouse tail. In contrast, DeepLabCut demonstrated greater robustness in these scenarios, providing a more complete and accurate localization of key points (as shown in Figure 3).

**Figure 4:**
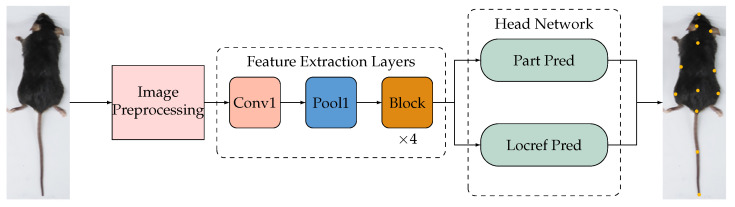
Overview of the DeepLabCut network architecture.

DeepLabCut’s flexibility allows for customization of tracking parameters tailored to specific experimental setups, extending its applicability to different animals and across large-scale experiments. Additionally, its architecture, containing feature extraction layers (FELs) and a head network, is optimally designed for feature extraction, followed by precise keypoint location prediction, as depicted in Figure 4.

Our investigation included six distinct network models for the FELs in DeepLabCut, namely ResNet-50, ResNet-101, ResNet-152 [44], EfficientNet-b0, EfficientNet-b3, and EfficientNet-b6 [45], which were trained using our collected experimental video data. The efficiency and precision of EfficientNet-b6 stood out, making it the optimal choice for DeepLabCut FELs within ABNet.

Moreover, the head network of DeepLabCut performs dual operations, namely “Part Pred” for body part prediction and “Locref Pred” for accurate key point positioning, utilizing the extracted features from the FELs for enhanced prediction accuracy.

## 4. Feature Extraction

After keypoint detection, a feature extraction module is required in ABNet to obtain information on mouse actions from the videos. The structure of this module influences not only the processing speed but also the accuracy of the subsequent clustering process. The spatial and temporal information of mouse actions is also important. For the above considerations, ST-GCN [21] is a nice choice to incorporate this information while simultaneously maintaining speed and accuracy. Based on ST-GCN and the task of ABNet, an enhanced method is proposed in this section.

### 4.1. Shortcomings of ST-GCN on Action Recognition Task

**Figure 5:**
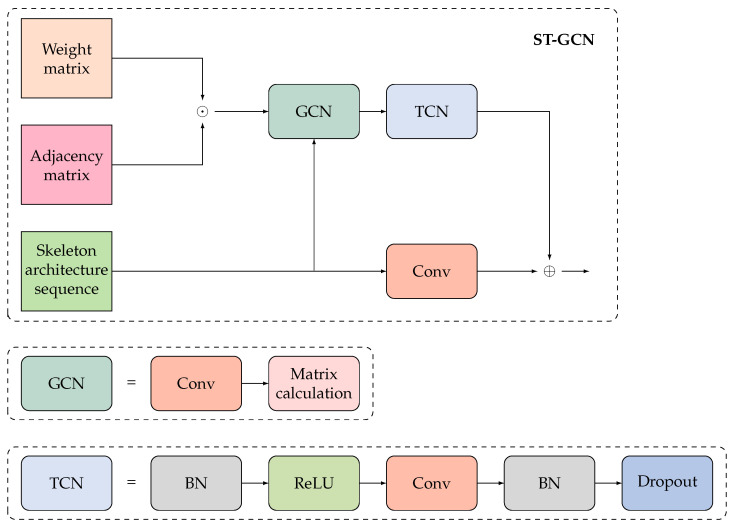
ST-GCN network structure.

Skeleton sequences provide temporal information about mouse behavior according to artificially defined rules, enabling the quantification of mouse behavior. However, these rules fail to automatically capture the spatial relationships between skeleton key points and dynamic features during movement. Given the unique structure of the skeleton, presenting the structural sequence information in the form of a graph is more suitable. Therefore, instead of relying on convolutional neural networks primarily designed for the processing of Euclidean data, we utilize ST-GCN as the feature extractor module, which effectively aggregates non-Euclidean domain information. The network architecture of ST-GCN is shown in Figure 5.

**Figure 6:**
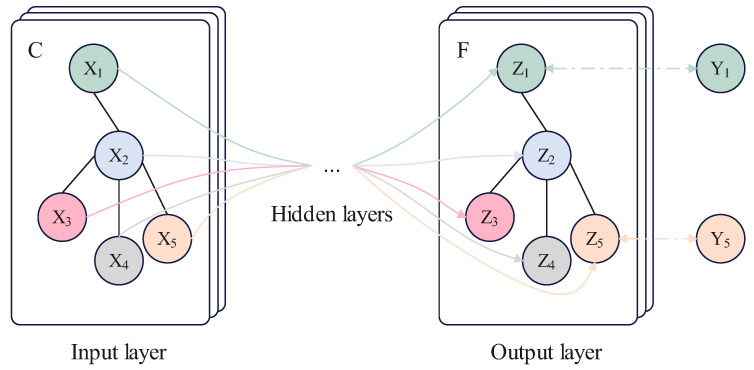
Diagram of GCN principles.

**Figure 7:**
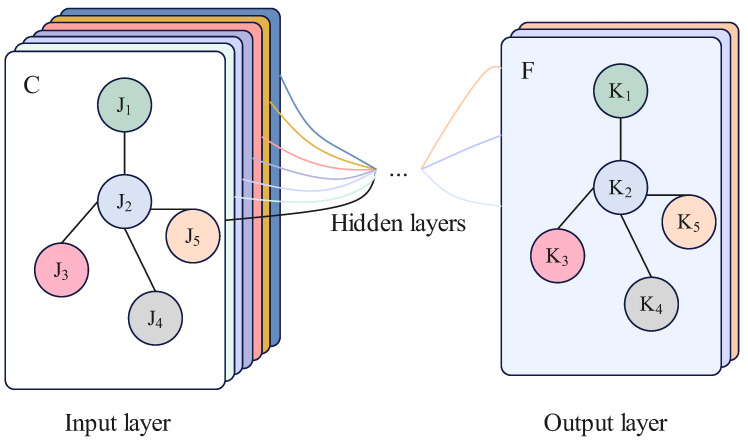
Diagram of TCN principles.

ST-GCN creates a comprehensive representation of skeleton sequence information by combining both spatial and temporal graph convolution by obtaining the weighted adjacency matrix, which is derived by multiplying the adjacency matrix by the bitwise generated weighted adjacency matrix. First, the matrix and skeleton sequence are fed into a Graph Convolution Network (GCN) to aggregate information in the spatial dimension of the input data. Then, it is passed into a Temporal Convolution Network (TCN) to aggregate information in the temporal dimension of the input data. Figure 6 and Figure 7 illustrate the principles of GCN and TCN, respectively.

However, the following shortcomings are associated with ST-GCN on mouse action recognition tasks:

- ST-GCN fails to transmit information on multiple connected key points. The predefined adjacency matrix of ST-GCN has a relatively simple structure, focusing only on the feature information of adjacent key points. As a result, it may yield unsatisfactory results when detecting behaviors involving multiple key points.
- ST-GCN ignores the influence of channel importance and adopts a default feature fusion strategy, which combines features from all channels during graph convolution. However, this approach overlooks the significance of individual channels and the influence of their relationships on the overall network performance. Take the feature mapping of the convolution operation (*F*), as an example (shown as Equation (1), where *X* is the input of shape $H^{\prime }\times W^{\prime }\times C^{\prime }$; *U* is the output of shape H×W×C; and *H*, *W*, and *C* are the height, width, and number of channels, respectively).
  (1) $$F:X\to U,\;X\in R^{H^{\prime }\times W^{\prime }\times C^{\prime }},\;U\in R^{H\times W\times C}$$
  When the convolution kernel ($V=[v_{1},v_{2},\cdots ,v_{n}]$) is applied, *U* can be represented by Equation (2), where $v_{c}^{s}$ is the 2D space kernel of a single channel on the corresponding channel ($v_{c}$) of *x*. Convolution operations sum up the convolution results from each channel, causing the channel-feature relationship to become intertwined with the spatial relationship learned through convolution.
  (2) $$U=\left[u_{1},u_{2},\cdots ,u_{H\times W}\right]:u_{c}=v_{c}*X=\sum_{s=1}^{C^{\prime }}v_{c}^{s}*x^{s}$$

### 4.2. Enhanced Approaches of ST-GCN

To improve ST-GCN and make the feature extraction module of ABNet more powerful, we propose several enhanced approaches to ST-GCN, including a Gaussian noise matrix and SENet.

#### 4.2.1. Gaussian Noise Matrix

The original ST-GCN is improved to enhance the feature extraction capability for mouse skeleton sequences. In the original ST-GCN, the graph convolution equation is shown as Equation (3).

(3) $$y=\Omega_{1\times 1}\left(XA\right)\cdot M\;\;A=\Lambda^{-\frac{1}{2}}A\Lambda^{-\frac{1}{2}}$$

In Equation (3), we have *k* as the number of key points. Then, *X* of shape 2×k is the location information of the key points, and the adjacency matrix, denoted as *A* of shape k×k, represents the connections between key points within a single frame. $\Lambda^{-1/2}$ represents a normalized diagonal matrix. *M* refers to a learnable weight matrix. The symbol $\Omega_{1\times 1}$ is a 1×1 convolution operation.

To deal with the limitations of the single adjacency matrix structure, we propose a perturbation mechanism utilizing Gaussian noise. This approach introduces Gaussian noise into the spatial graph convolution layer of the original ST-GCN. Adding noise to the original adjacency matrix creates a new adjacency matrix, which allows for connections between non-adjacent skeleton key points, thereby obtaining a more appropriate spatial structure to describe action samples and enhance detection accuracy. The Gaussian noise matrix (*G*) shares a similar structure with $A_{k}$, shown as Equation (4), where the matrix (φ) follows a normal distribution, with μ as the mean and σ as the variance.

(4) $$G=\Lambda \cdot \varphi ,\;\varphi ∼N(\mu ,\sigma^{2})$$

The equation for graph convolution undergoes modifications upon the introduction of the Gaussian noise matrix (*G*), which is shown as Equation (5).

(5) $$y=\Omega_{1\times 1}\left(XA+G\right)\cdot M$$

#### 4.2.2. Squeeze and Excitation

**Figure 8:**
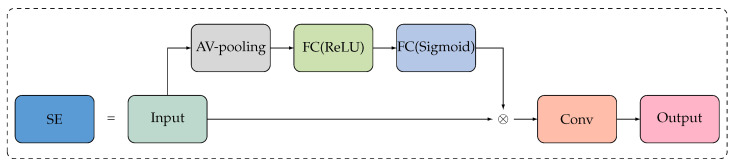
Overview of the SE module structure.

When performing graph convolution, ST-GCN fuses all channels’ characteristics, ignoring the impact of channel importance and channel relationship on network performance. To enable ST-GCN to prioritize channel features with the most informative content while suppressing less important ones, we introduced the Squeeze-and-Excitation (SE) module to ST-GCN. The SE module is shown in Figure 8.

The SE module incorporates the squeeze and excitation operations, allowing for the separation of channel features, which makes the model directly learn channel relationships. The squeeze operation, shown as Equation (6), encodes spatial features within channels into a global feature, addressing the issue of limited extraction of channel relationship information caused by the local space constraint of convolution. *z* is the channel information and is *C*-dimensional, and $u_{c}$ is the feature map for input.

(6) $$z_{c}=F_{sq}\left(u_{c}\right)=\frac{1}{H\times W}\sum_{i=1}^{H}\sum_{j=1}^{W}u_{c}\left(i,j\right),z\in R^{C}$$

The excitation operation, shown as Equation (7), allows us to capture the dependency relationships among channels within the global feature information aggregated by the squeeze operation. It utilizes a gate mechanism with a sigmoid activation function, enabling the SE module to learn non-linear interactions between channels and emphasize the significance of multiple channels rather than just individual ones.

(7) $$s=F_{ex}\left(z,\Omega \right)=\sigma \left(g\left(z,\Omega \right)\right)=\sigma \left(\Omega_{2}\mathrm{ReLU}\left(\Omega_{1}z\right)\right)$$

In Equation (7), $\Omega_{1}\in R^{\frac{C}{r}\times C}$, and $\Omega_{2}\in R^{\frac{C}{r}\times C}$. To enhance the model’s generalization ability, the excitation operation of the SE module incorporates two fully connected (FC) layers to parameterize the gate control. The first FC layer reduces the dimensionality of the feature information, which is then restored to the original dimension through a ReLU activation function and another FC layer. The equation for this process is shown as Equation (8), where $\tilde{X}=\left[{\tilde{x}}_{1},{\tilde{x}}_{2},\cdots ,{\tilde{x}}_{c}\right]$ and $F_{scale}\left(u_{c},s_{c}\right)$ refers to channel-wise multiplication between the scalar ($s_{c}$) and the feature map ($u_{c}\in R^{H\times W}$).

(8) $${\tilde{x}}_{c}=F_{scale}\left(u_{c},s_{c}\right)=s_{c}u_{c}$$

**Figure 9:**
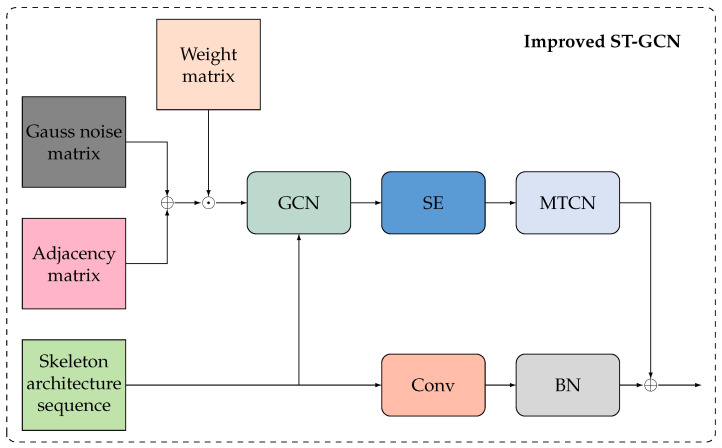
Overview of the improved ST-GCN network.

The model can capture correlations between non-adjacent key points by perturbing the adjacency matrix with Gaussian noise. Additionally, incorporating feature compression and activation operations from the SE module allows the model to learn inter-channel relations with only a slight increase in computation, ultimately enhancing detection performance. Figure 9 shows the enhanced ST-GCN model.

#### 4.2.3. Multi-Branch TCN

ST-GCN uses a 1×1 convolution with a kernel size of 9 for modeling in the time dimension. The large kernel covers a wide receptive field. However, this design lacks flexibility, resulting in both calculation and parameter redundancy.

**Figure 10:**
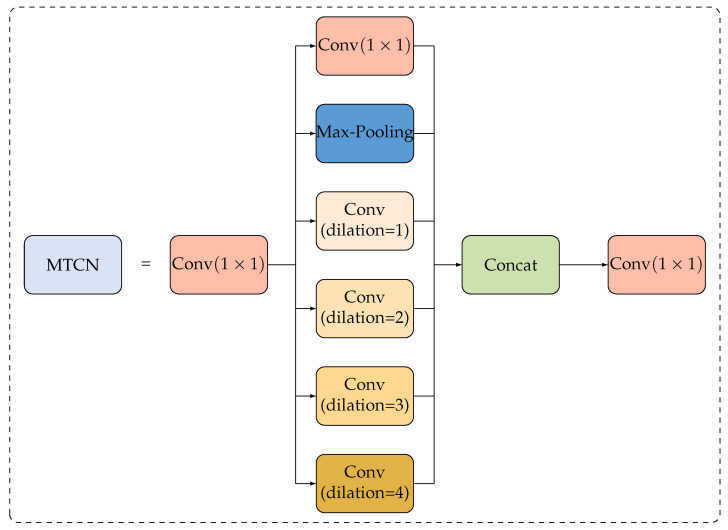
Overview of multi-branch TCN.

We use a multi-branch temporal convolution network (MTCN) instead of a single-branch design, as shown in Figure 10. MTCN has six branches, namely a 1×1 conv branch, a max pooling branch, and four 1D time-domain conv branches with a 3×3 kernel and an expansion range of 1 to 4. The 1×1 conv is first applied to convert features and divides them into six groups with the same channel width. Then, each feature group is processed with a separate branch. The six outputs are connected and processed by another 1×1 conv to obtain the output of MTCN. It not only improves the time modeling ability but also saves computational cost and parameters, as the channel width of each branch is reduced.

## 5. Behavior Analysis

In this section, we propose a method for abnormal behavior detection using clustering, which is based on the classification results of general behavior detection. The training methods and input data are also introduced.

### 5.1. General Behavior Analysis

After extracting features from a video of mouse movement using Enhanced ST-GCN, normal behavior prediction is performed with a classification approach. During the training process, the dataset is derived from our annotated videos, where each normal action consists of a short video segment lasting 3 s. After obtaining the skeletal structure sequence with DeepLabCut, this sequence is input into the Enhanced ST-GCN network. The network consists of multiple spatio-temporal graph convolution operation layers, followed by batch normalization, global pooling, and a softmax classifier. The purpose of the Enhanced ST-GCN is to detect general behaviors. For abnormal behaviors, a clustering method is applied.

### 5.2. Abnormal Behavior Detection

Deep neural networks combined with a classifier are commonly used to recognize mouse behavior. However, the classifier method is an appropriate choice only when the behavior of mice can be fully defined during the observation of medical experiments. In medical experiments, it is often necessary to recognize all the actions and behaviors of mice, and some behaviors cannot be defined in advance.

Therefore, to effectively identify the abnormal behavior of mice, we employ the approach of clustering instead of classification as the downstream task. It involves principal component analysis combined with DBSCAN [46], a clustering algorithm, to extract correlations between different feature information encoded by ST-GCN. Based on these correlations, the feature information is divided into distinct behavior clusters. Feature points that do not belong to any cluster are considered indicators of abnormal behavior.

The feature information extracted by neural networks often has large dimensions, which is associated challenges in effectively clustering this kind of data. To enhance the effect of clustering, we employ principal component analysis (PCA) to transform the high-dimensional data into a lower-dimensional representation, which helps improve clustering accuracy and reduces computational complexity.

DBSCAN stands out from clustering algorithms such as K-means, as it eliminates the need to specify the number of clusters in advance. This is particularly beneficial in medical experiments, where the manifestation of abnormal behaviors by mice cannot be predetermined. In ABNet, we extract feature information, apply dimensionality reduction techniques, and feed the processed data into the DBSCAN algorithm for clustering. This yields distinct clusters, with each cluster representing a normal behavior. Outliers that do not belong to any cluster are indicative of abnormal behavior. Biologists or experimenters will subsequently define the behaviors associated with these outlier points.

## 6. Experiment

In this section, we conduct extensive experiments to demonstrate the effectiveness of ABNet. First, in Section 6.1, we describe the experimental setup, which is the foundation for smoothly conducting the experiments. Our experiments are conducted in a robust computational environment using training servers equipped with high-performance CPUs and GPUs to ensure the model operates in optimal configurations. Alongside this, detailed descriptions of the model training parameters, such as learning rate, batch size, and training epochs, are provided to ensure the reliability and reproducibility of the experiments. Next, in Section 6.2, we introduce the datasets and evaluation metrics used in our experiments. Subsequently, in Section 6.3, we conduct a series of ablation experiments to assess the effectiveness of different components when optimizing ST-GCN. Following that, in Section 6.4, we perform comparative experiments on the Kinetics-Skeleton dataset, comparing our improved ST-GCN model with various behavior recognition algorithms. Lastly, in Section 6.5, we validate the superior performance of ABNet in detecting abnormal mouse behaviors. The results highlight the outstanding ability of our model to extract and encode motion information.

### 6.1. Experimental Setup

We established a model-training server configured with a 2.1 GHz Intel(R) Xeon(R) Platinum 8352V CPU and 8 RTX 4090 GPUs. The server has CUDA 12.1 and PyTorch 2.0.0 installed. We used the SVG optimizer with an initial learning rate of 1 × 10^−1^. The batch size is 64, and the training runs for 100 epochs. Training is conducted using stochastic gradient descent, and to prevent overfitting, data augmentation techniques such as scaling and translation are employed. The scaling factor is randomly chosen between 0.97 and 1.03, and the translation randomly shifts pixels between −5 and 5 pixels. The model’s input consists of skeleton sequence data with dimensions of N, C, T, V, and M, where N represents the number of videos, C represents the joint features, usually consisting of 3 features per joint, namely x, y, and acc (confidence level). Here, x and y are the positional coordinates of the joint, and acc is the confidence level. T represents the maximum number of key frames. V is the number of joints, with 12 joints annotated in this experiment. M represents the number of targets per frame, with M set to 1 for detection of a single target animal in this experiment.

### 6.2. Dataset and Evaluation Metrics

Kinetics-Skeleton Dataset [47] is a large-scale human motion dataset comprising 300,000 video clips from 400 different categories. These video clips are sourced from a diverse range of YouTube videos. Each clip in Kinetics has a duration of approximately 10 s, with a total of either 30 or 300 frames per second (FPS). However, the dataset only provides the raw video clips without any skeleton information. To address this, ST-GCN utilizes the publicly available OpenPose [48] toolkit to estimate the positions of 18 joints in each frame of the clips. This process converts a clip with T frames into a sequence of skeletons represented by multiple tuples, resulting in the creation of the Kinetics-Skeleton dataset. The dataset is divided into a training set with 240,000 clips and a validation set with 20,000 clips. Following the evaluation methodology described in [47], we train our models on the training set and report the top-1 and top-5 accuracy on the validation set.

Top-1 and top-5 accuracy are common evaluation metrics used to assess the performance of classification models, particularly in scenarios with a large number of classes, such as image or video classification tasks.

Top-1 accuracy measures the percentage of samples for which the model’s highest probability prediction matches the true class label. It is a strict measure, as it considers only the single most likely prediction.

(9) $$\mathrm{Top}-1\;\mathrm{Accuracy}=\frac{\mathrm{Number}\;\mathrm{of}\;\mathrm{Correct}\;\mathrm{Predictions}}{\mathrm{Total}\;\mathrm{Number}\;\mathrm{of}\;\mathrm{Samples}}$$

Top-5 accuracy is more lenient and determines how often the true class label is among the top-five predictions (those with the highest probabilities) of the model. This is particularly useful in tasks with many similar classes, as it allows for some flexibility in evaluating the model’s predictions.

(10) $$\mathrm{Top}-5\;\mathrm{Accuracy}=\frac{\mathrm{Number}\;\mathrm{of}\;\mathrm{Samples}\;\mathrm{with}\;\mathrm{True}\;\mathrm{Class}\;\mathrm{in}\;\mathrm{Top}\;5\;\mathrm{Predictions}}{\mathrm{Total}\;\mathrm{Number}\;\mathrm{of}\;\mathrm{Samples}}$$

These metrics provide insights into how well the model performs not only at identifying the single best class (top-1) but also how robust its predictions are in terms of ranking potential class candidates (top-5). Together, they offer a comprehensive view of a model’s classification performance.

**The mouse behavior dataset** was collected by ourselves to evaluate and compare the detection performance of different types of behavior recognition methods. It contains 10 h of real monitoring video data from life science research for our experiments. First, we define the following four distinct actions for mouse behavior: (1) movement, which refers to straight-line locomotion; (2) head turning, where the body remains still or exhibits minimal rotation while the head turns at a significant angle; (3) standing, which involves the mouse supporting itself with its front paws on a side barrier; and (4) turning, which indicates a change in the linear angle of the entire spinal column. Subsequently, we crop the videos every 50 frames and annotate them accordingly. Finally, we randomly split the dataset into training and testing sets using an 8:2 ratio.

In this paper, we employ the Average Precision (AP) metric to evaluate the performance of our model. AP is a widely recognized evaluation measure in abnormal action recognition tasks, providing a comprehensive assessment by considering both precision and recall. The AP is calculated by integrating the Precision–Recall (PR) curve as follows:

(11) $$\mathrm{AP}=\int_{0}^{1}p\left(r\right)\;dr$$

In this equation,

- p(r) represents the precision at a given recall level (*r*);
- *r* denotes the recall, which varies between 0 and 1.
  Precision (Precision) and recall (Recall) are defined by the following expressions:
  (12) $$\mathrm{Precision}=\frac{TP}{TP+FP}$$
  (13) $$\mathrm{Recall}=\frac{TP}{TP+FN}$$
  where
- TP (true positives) indicates the number of correctly identified positive instance;
- FP (false positives) indicates the number of incorrectly identified positive instances.
- FN (false negatives) indicates the number of actual positive instances that are incorrectly identified as negative.

The precision–recall curve is plotted to calculate AP, and the area under this curve is computed using numerical integration methods. This approach allows for the evaluation of the model’s detection accuracy across various decision thresholds.

### 6.3. Ablation Experiment of Enhanced ST-GCN

This section uses the Kinetics-Skeleton dataset as a benchmark, mainly because of its diversity in action categories. By training on a training set of 240,000 video clips and validating on a validation set of 20,000 clips, we can effectively validate the feature extraction capabilities of our proposed model. The complexity and challenge of this dataset provide an ideal platform for assessing the model’s performance in handling complex spatio-temporal behavior patterns. Furthermore, using such a versatile dataset helps better demonstrate the potential applicability of our model to cross-species behavior recognition tasks. We also conduct an in-depth evaluation of the performance differences between the original ST-GCN model and our enhanced version of the Kinetics-Skeleton dataset. We analyze how different enhancements contribute to the overall performance. The results are summarized in Table 1.

Our enhancements aim to improve the model’s feature extraction and encoding capabilities by capturing correlations between non-adjacent skeletal key points and emphasizing essential channel features. As shown in the table, adding Gaussian noise (Gauss + ST-GCN) improves performance, with the top-1 accuracy rising from 30.7% to 31.2% and the top-5 accuracy increasing from 52.8% to 53.3%. This suggests that Gaussian noise augments the skeletal spatial structure and enhances detection accuracy at a relatively low computational cost.

The SE module (SE + ST-GCN) results in further improvements, achieving a top-1 accuracy of 31.4% and a top-5 accuracy of 54.0%. This indicates that the SE module effectively helps the model focus on informative channel features, significantly boosting overall detection performance.

The combination of Gaussian noise and the SE module (Gauss + SE + ST-GCN) provides even more substantial enhancements, with a top-1 accuracy of 32.4% and top-5 accuracy rising to 54.7%. This demonstrates the synergistic effect of combining these components, suggesting that they address each other’s limitations and enhance the model’s ability to learn complex, high-value features.

Finally, we achieved the highest performance by incorporating an additional enhancement with the MTCN module (Gauss + SE + MTCN + ST-GCN), with top-1 accuracy climbing to 32.7% and top-5 accuracy further increasing to 55.2%. This demonstrates improved temporal modeling capabilities and highlights the potential for optimizing parameters to reduce computational costs while achieving significant performance gains. The comparative analysis demonstrates that our enhanced ST-GCN model significantly outperforms the original ST-GCN, highlighting the effectiveness of integrating multiple key components to enhance feature extraction and encoding capabilities. Good feature extraction and encoding capabilities can help researchers achieve more accurate behavior classification when analyzing normal mouse behavior. When analyzing abnormal behavior, these capabilities allow for better modeling of normal behavior samples, resulting in superior and more representative features. This enables clustering algorithms to more accurately distinguish abnormal behavior.

### 6.4. Comparison Experiments of Enhanced ST-GCN

In the comparison experiments, our goal was to validate the capability of the improved ST-GCN model in extracting and encoding motion information. We conducted a comparative analysis on the Kinetics-Skeleton dataset with several other behavior recognition algorithms, namely feature encoding [49], Deep LSTM [50], Res-TCN [51], the original ST-GCN [21], CoST-GCN, CoAGCN, and CoS-TR [52]. The experimental results are shown in Table 2.

The table demonstrates that our enhanced ST-GCN model achieves superior detection accuracy on the Kinetics-Skeleton dataset compared to the other examined algorithms. The feature encoding method represents each video frame using existing features, extracts additional temporal features, and uses ranking machine learning to order the frames for a new video representation. Although this method can capture temporal dynamics, the necessity of training separate ranking functions for each action category, combined with inherent limitations of ranking models, constrains its accuracy, as reflected in its top-1 accuracy of 14.9% and top-5 accuracy of 25.8%. The Deep LSTM algorithm utilizes a recurrent neural network (RNN) structure to group skeleton key points based on spatial context. It processes these groups using LSTM units, aiming to effectively learn sequential dependencies. However, it needs help in efficiently capturing temporal features over extended sequences, resulting in relatively low accuracy rates of 16.4% for top-1 and 35.3% for top-5. The Res-TCN model employs stacked convolution units within temporal convolutional networks (TCNs) to enhance interpretability and manage temporal attention. Despite achieving better results, with a top-1 accuracy of 20.3% and a top-5 accuracy of 40.0%, the lack of an attention mechanism to fine tune temporal focus limits its performance advancements. The original ST-GCN serves as a baseline, leveraging the natural topology of skeleton key points for action recognition and achieving a top-1 accuracy of 30.7% and top-5 accuracy of 52.8%, showing promising results but also leaving room for improvement in temporal representation.

Additional methods, such as CoST-GCN, CoAGCN, and CoS-TR, offer varying performance against the original ST-GCN and our model, achieving top-1 accuracies of 32.2%, 27.5%, and 29.9%, respectively. Our enhanced ST-GCN (Gauss + SE + MTCN) builds upon the original by integrating Gaussian perturbations, channel attention mechanisms, and a multi-branch TCN structure. These enhancements allow the model to better focus on critical temporal segments and optimize feature mapping, leading to improved performance, with a top-1 accuracy of 32.7% and a top-5 accuracy of 55.2%.

Overall, the improved ST-GCN demonstrates significant performance gains, particularly in its ability to preserve and leverage the inherent information of skeletal structures while better addressing temporal dynamics, ultimately achieving the highest accuracy among the compared methods.

### 6.5. Comparative Evaluation of Abnormal Behavior Recognition in Mice

In order to evaluate the effectiveness of our proposed method for recognizing abnormal actions in mice, we conducted a series of comparative experiments. These experiments involved established algorithms such as GEPC [42], TSC [41], Conv + LSTM [53], VideoMAE V2 [54], TAdaConvNeXtV2-B [55], and CAST-B/16 [56], utilizing a comprehensive mouse behavior dataset. The performance results for these algorithms are systematically outlined in Table 3.

Table 3 demonstrates the superior performance of our method in the field of mice mouse action recognition. Specifically, our method achieved an average precision (AP) of 83.1%, significantly surpassing the other methods. The TSC algorithm achieved an AP of 79.5%, while GEPC achieved 75.2% and Conv + LSTM achieved 75.6% accuracy. These results highlight the distinct accuracy advantage of our approach.

The TSC algorithm focuses on recognizing anomalies through behavior reconstruction. It learns the dynamic features of normal behaviors, reconstructs the observed behavior based on these features, and identifies anomalies by measuring the reconstruction error. However, due to the ability to reconstruct both normal and abnormal behaviors, it occasionally results in false negatives due to its limited robustness.

In contrast, the GEPC algorithm bypasses the motion feature extraction phase and directly employs clustering on skeletal data to segregate normal and abnormal behaviors. While straightforward, this technique can lead to false positives and negatives because it relies solely on skeletal data for clustering.

Methods like Conv + LSTM, TAdaConvNeXtV2-B, CAST-B/16, and VideoMAE V2 rely on training with anomalous behavior samples. When these anomalies exhibit significant variance and scarcity, these methods struggle to model abnormal behavior, leading to poor detection performance. Such methods may perform well when anomalous samples are abundant and representative, but their performance diminishes when samples are scarce.

Our proposed method addresses these limitations through a distance-based model. It first applies principal component analysis (PCA) to reduce the dimensionality of high-dimensional feature sets extracted from the ST-GCN model. These reduced features are then subjected to clustering algorithms, where normal behaviors form tight clusters and deviations from these clusters are readily identified as anomalies.

**Figure 11:**
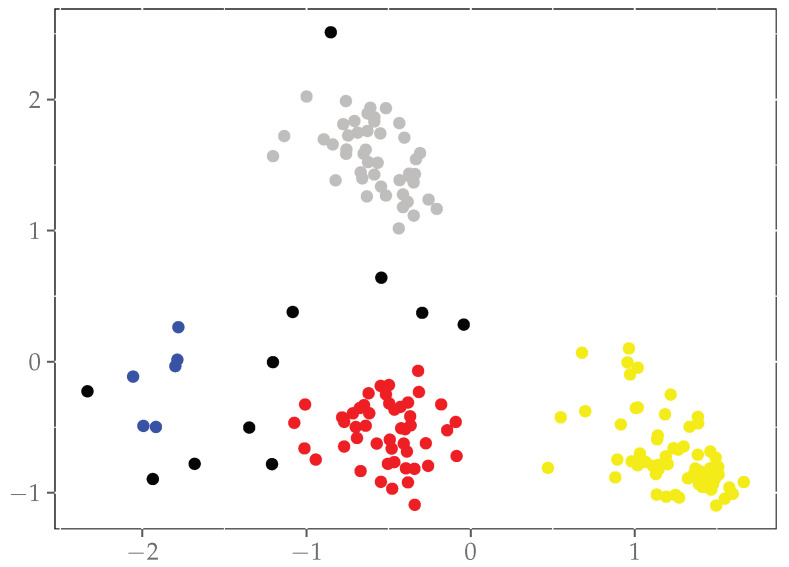
Visualization of clustered feature information, with black outliers identified as abnormal behavior. The red dots represent movement, the blue dots represent turning, the yellow dots represent standing, the gray dots represent head turning, and the black dots represent abnormal behavior.

The model’s effectiveness lies in its robust high-dimensional features specific to normal behavior, capturing comprehensive motion information and providing clear distinctions between normal and abnormal characteristics. As a result, it significantly reduces false positives and false negatives, enhancing detection accuracy compared to other algorithms. The clustering outcomes are visually represented in Figure 11, where outliers are marked by black dots, signifying detected abnormal behaviors.

## 7. Conclusions

In this paper, we propose ABNet to identify and analyze abnormal mouse behavior. ABNet utilizes an enhanced ST-GCN as a general behavior analysis module, incorporating Gaussian noise matrices, SE modules, and multi-branch TCN structures to bolster the ST-GCN model. The improved model extracts and encodes motion features from mouse skeleton sequences. Subsequently, principal component analysis (PCA) is applied to reduce the dimensionality of the feature information. Finally, clustering is used to differentiate abnormal behaviors. Experimental results demonstrate that our method achieves superior performance in accurately identifying and distinguishing abnormal mouse behaviors compared to existing methods. The high accuracy of ABNet in identifying abnormal mouse behavior helps researchers monitor and analyze mouse behavior more precisely, reducing misjudgments and improving the reliability of experimental results.

Furthermore, since ABNet does not rely on sensors or biomarker analysis, it minimizes physical interference with the mice, allowing them to exhibit behavior in a more natural state, thereby obtaining more authentic experimental data. Additionally, the efficiency and automation of ABNet significantly reduce the time and cost of manual monitoring and analysis, allowing researchers to focus more on experimental design and data interpretation. Finally, the high accuracy and non-invasiveness of ABNet give it potential for applications in a broader range of experimental environments beyond neuroscience, including pharmacology and toxicology.

## 8. Future Work

In this paper, we explored the effectiveness of using semi-supervised deep learning methods to detect abnormal behavior in laboratory animals while noting the significant advantages of multimodal biometric systems in enhancing recognition accuracy and security [57]. Future research can leverage the strengths of multimodal systems by integrating various behavioral features to improve detection performance and optimizing deep learning algorithms to enhance processing speed and accuracy. For example, behavior recognition and anomaly detection can be enhanced through the use of multimodal approaches such as combining audio data with image data, integrating near-infrared data with conventional video images, merging radar data with image data, and combining electroencephalogram (EEG) and motion sensor signals with image data. Additionally, incorporating defense mechanisms from multimodal systems may help increase the robustness of abnormal behavior detection. Research should also consider the practical needs of the laboratory environment, gradually incorporating multimodal features and exploring the application of this technology in various experimental settings to overcome the current method’s limitations and advance the development of laboratory animal behavior detection technology.

## Figures and Tables

**Table 1:** Performance of ST-GCN with different component combinations. Gauss represents the Gaussian noise matrix, SE stands for the squeeze-and-excitation module, and MTCN denotes the Multi-Branch TCN.

Method	Top-1 (%)	Top-5 (%)
ST-GCN	30.7	52.8
Gauss + ST-GCN	31.2	53.3
SE + ST-GCN	31.4	54.0
Gauss + SE + ST-GCN	32.4	54.7
Gauss + SE + MTCN + ST-GCN	32.7	55.2

**Table 2:** Comparison of action recognition algorithms on the Kinetics-Skeleton dataset, show casing top-1 and top-5 accuracy rates.

Method	Top-1 (%)	Top-5 (%)
Feature Encoding	14.9	25.8
Deep LSTM	16.4	35.3
Res-TCN	20.3	40.0
ST-GCN	30.7	52.8
CoST-GCN	32.2	54.5
CoAGCN	27.5	49.1
CoS-TR	29.9	52.7
Ours	32.7	55.2

**Table 3:** Comparison of abnormal behavior detection algorithms using mouse behavior dataset.

Method	Average Precision (AP) (%)
TSC	79.5
GEPC	75.2
Conv + LSTM	75.6
VideoMAE V2	81.9
TAdaConvNeXtV2-B	81.4
CAST-B/16	81.1
Ours	83.1

## Data Availability

The data included in this study may be provided upon request to the corresponding author due to ethical and privacy protection restrictions.

## Abbreviations

The following abbreviations are used in this manuscript:

AI	Artificial Intelligence
AP	Average Precision
FC	Fully Connected
FEL	Feature Extraction Layer
FPS	Frames Per Second
GAN	Generative Adversarial Network
GCN	Graph Convolution Network
MTCN	Multi-Branch Temporal Convolution Network
n-D	n-Dimension
PCA	Principal Component Analysis
RNN	Recurrent Neural Network
SE	Squeeze and Excitation
ST-GCN	Spatio-Temporal Graph Convolution Network
SVM	Support Vector Machine
TCN	Temporal Convolution Network
